# Supplementary material for: Identification of Potential Therapeutic Targets for Sensorineural Hearing Loss and Evaluation of Drug Development Potential Using Mendelian Randomization Analysis
Source: Bioengineering (Basel). 2025 Jan 29;12(2):126. doi: 10.3390/bioengineering12020126 (PMC11852220; doi:10.3390/bioengineering12020126)
Supplement: Supplementary file 1 [file bioengineering-12-00126-s001.zip › bioengineering-3419918-supplementary/Supplementary materials/Supplementary Material 3.pdf]

Table 1 . Colocalization analysis results of plasma proteins LATS1, TEF, LMNB2, OGFR, and EIF2AK3 with SNHL.

Proteins	SNPs	PPH0	PPH1	PPH2	PPH3	PPH4	PPH3+PPH4
LATS1	9294	2.32E-12	0.053132917	1.08E-11	0.246791825	0.700075259	0.946867083
TEF	6193	1.92E-15	0.018015474	7.38E-14	0.692654832	0.289329694	0.981984526
LMNB2	10247	5.56E-05	0.088782896	5.43E-05	0.085956184	0.825151048	0.911107232
OGFR	10875	1.42E-37	0.01346408	1.04E-35	0.986220853	0.000315067	0.98653592
EIF2AK3	3717	0.7415516	0.164741138	0.06874449	0.015262395	0.009700346	0.024962741
