# Supplementary material for: Identification of Potential Therapeutic Targets for Sensorineural Hearing Loss and Evaluation of Drug Development Potential Using Mendelian Randomization Analysis
Source: Bioengineering (Basel). 2025 Jan 29;12(2):126. doi: 10.3390/bioengineering12020126 (PMC11852220; doi:10.3390/bioengineering12020126)
Supplement: Supplementary file 1 [file bioengineering-12-00126-s001.zip › bioengineering-3419918-supplementary/Supplementary materials/Supplementary Material 6.pdf]

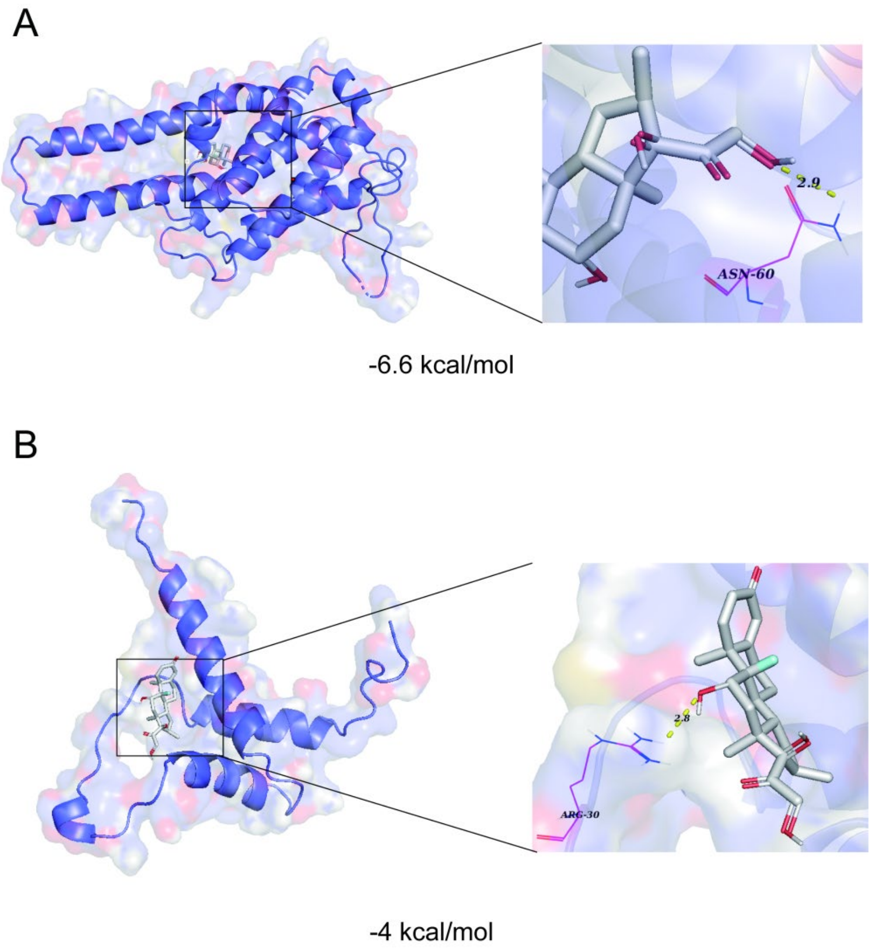

Figure S1. Molecular docking results for dexamethasone and target proteins.(A) LATS1 docking with dexamethasone , (B) TEF docking with dexamethasone .
